# Effect of Alloying Additives and Moulding Technology on Microstructure, Tightness, and Mechanical Properties of CuSn10 Bronze

**DOI:** 10.3390/ma16247593

**Published:** 2023-12-11

**Authors:** Dawid Witasiak, Aldona Garbacz-Klempka, Marcin Papaj, Piotr Papaj, Maria Maj, Marcin Piękoś, Janusz Kozana

**Affiliations:** 1 Faculty of Foundry Engineering, AGH University of Science and Technology, Reymonta 23, 30-059 Krakow, Poland; witasiak@agh.edu.pl (D.W.); mmaj@agh.edu.pl (M.M.); mpiekos@agh.edu.pl (M.P.); jkozana@agh.edu.pl (J.K.); 2 Zakład Odlewniczy METAL-KOLOR sp. z o.o., Ostrowiecka 5, 27-200 Starachowice, Poland; marcin@metalkolor.pl (M.P.); piotr@metalkolor.pl (P.P.)

**Keywords:** bronzes, Cu-Sn alloys, tin, cast alloys, casting tightness, casting microstructure, material characterisation, mechanical properties, SEM-EDS

## Abstract

Thise research was conducted to determine the impact of the applied casting technology, mould and alloying additives on the tightness of the CuSn10 cast alloy. Under industrial conditions, a series of experimental melts were made that were characterised by varying the concentrations of the main alloying element (Sn) and the introduced alloying additives (Si, Zn, Zr). The mould was made from green sand and used the CO_2_ moulding process. To assess the influence of the alloying additives, a metallographic analysis of the studied alloy was carried out, and the alloy’s microstructure was examined using optical and scanning electron microscopy. The introduced alloying additives affected the properties and microstructure of the studied alloy. As alloying additives, zirconium resulted in a visible refinement of the microstructure, while silicon improved the fluidity and quality of the casting’s external surface. The use of alloying additives and moulds made using different technologies is intended to improve the structure of the tin bronze castings produced and to find the best solution to significantly eliminate the lack of leakage of the castings. The castings were subjected to mechanical processing, and a leak test was performed using the pressure drop method. The conducted research allowed us to determine which technology, applied to production, will bring about a reduction in the problem and will inform further investigations.

## 1. Introduction

Tin bronzes belong to the group of copper alloys, which have the longest history [1,2,3]. Their functional properties were discovered in prehistoric times, giving a name to an entire era—the Bronze Age [4,5]. Due to their excellent mechanical properties resulting from the addition of tin to copper, tin bronzes were used for the production of tools and weapons, among other applications [6,7].

Due to their properties, including corrosion resistance, these bronzes currently have a significant presence in technology [8,9,10,11,12]. These alloys have found applications in automotive, electronics, and maritime industries, among others [13,14,15,16]. Thanks to their aesthetic appeal and corrosion resistance, tin bronzes are also used for creating sculptures, artistic works [17,18,19,20], and architectural elements.

The tin content in bronze alloys (intended for plastic processing) is up to 12% by weight, while the tin content is approximately 10% when casting tin bronzes [2,21,22,23,24]. 

The technological, strength, and plastic properties of the discussed bronzes are dependent on the amount of tin they contain [2,10,25,26,27]. In the green-sand-casting process of bronzes, it can be observed that, as the tin content increases, the values of the strength properties and hardness increase (with a simultaneous decrease in elongation). In the case of die-castings, tin contents of up to about 4% lead to increases in elongation; this decreases after crossing the 4% threshold. The impact strength of the CuSn alloy increases with tin contents of up to 5% Sn; however, it also decreases beyond that value. The tensile strength of tin bronzes increases with increases in tin content of up to 10%. The hardness of the alloy increases linearly with increases in the tin content in the alloy [2,13,28,29,30].

The microstructure of the CuSn alloy is closely related to the tin content in the alloy. In alloys where the Sn content is below 6%, the structure consists of a solid solution α. However, when the tin content is higher than 6%, the microstructure consists of a solid solution of Cu (α) that is rich in copper and eutectoid (α + δ). Due to their wide solidification range, CuSn alloys are susceptible to microsegregation during solidification. The axes and arms exhibit reduced tin content, while the interdendritic spaces are enriched in tin [31,32,33,34].

Zirconium introduced into the CuSn10 alloy exhibits a modifying effect on the alloy, including the refinement of its structure. The introduced modifier forms numerous intermetallic phases with the alloying components [35,36,37].

The most commonly added elements to tin bronze are lead and zinc. The addition of lead improves its machinability, sliding properties, and pressure tightness. This is especially important in high-lead bronzes, where lead (about 1%) seals the interconnected porosities [2,20]. Zinc added to tin bronze has a positive impact on its properties: it increases its fluidity, reduces the gas absorption by the alloy, acts as a deoxidiser, and increases the alloys conductivity. The addition of zinc [38] leads to the refinement of the alloy’s structure, which in turn positively affects the tightness of thin-walled castings [39]. Zinc in the alloy widens the solidification temperature range, which is an unfavourable phenomenon: it also negatively affects the sliding properties of the alloy [40].

Tin bronzes are among the most expensive bronze alloys. Their high cost is deter-mined by the deficit of the main alloying component tin, which has led to research that is aimed at reducing the tin content without losing the properties of the bronzes, by introducing other alloying elements [41,42]. Due to their broad and versatile properties, particularly high wear resistance and low friction coefficient, castings made from tin bronzes are used as parts in machinery that is subjected to heavy mechanical loads and parts that require high wear resistance [43,44]. Items such as worm gears, bearings, and bushings are made from this type of bronze [45,46,47,48]. Other applications include valves, armatures, and bells [20], as well as products that maintain pressure at temperatures of up to 260 °C.

In industrial applications, pressure tightness is essential for tin bronzes [49,50,51,52,53,54]. One of the drawbacks of tin bronzes is their tendency to form structural defects such as porosity. Tin significantly widens the solidification temperature range of bronzes, which is why tin bronzes have a tendency to form a dendritic structure during solidification [22]. Alloys with such a wide range of solidification temperatures create an isolated zone called the freezing sphere during solidification, resulting in the formation of interdendritic shrinkage or microporosity. The microporosity in tin bronzes is scattered throughout such castings with a tendency to cluster in the last solidifying areas of the castings [2]. Castings made of tin bronzes typically have porosities that range from 1 to 2%, and only small castings have porosities below 1%. To overcome this effect, it is crucial to design the casting process properly in terms of the gate system and the cooling. To achieve better results in this regard, it is important to ensure the rapid solidification of the castings. For relatively large castings with thicker walls, directional solidification is recommended, while uniform solidification is preferred for small, thin-walled castings [55,56]. 

In previous studies, the authors [11,12,13,15,29,35,37,42,48] have usually focused on determining the proportion of tin in the CuSn10 alloy, as well as the influence of alloying additives and modifiers on the mechanical properties of tin bronzes. Here, the research has been extended, as this paper considers the simultaneous influence of several factors, related both to the tin content and selected additives in tin bronzes, but also to the mould technology, including the moulding and core sand used. In doing so, not only are the mechanical properties considered, but also mainly the influence of the aforementioned factors on the tightness of castings produced under industrial conditions. Improving the quality of production is the main objective of application-oriented research.

## 2. Materials and Methods

Research that was aimed at determining the impact of the chemical composition and casting technology on the tightness of castings made from tin bronzes was conducted under industrial conditions at the METAL-KOLOR Foundry and at the Faculty of Foundry of AGH University of Science and Technology in Krakow. The influence of the concentration of the main alloying elements and additives was studied using a base CuSn10 alloy.

The additives that were introduced into the alloy were selected based on their own experimental research and the available literature.

### 2.1. Preparation of Alloys

The quality of produced castings is significantly influenced by the charge material. Contaminants in the charge material can lead to the formation of harmful gases, especially hydrogen. The most common contaminants in the charge material include greases, oils, and emulsions. To avoid the defects that are caused by low-quality charge materials, special attention should be paid to their origin and storage.

For the purpose of our own research, a CuSn10 casting alloy was prepared according to EN 1982 [57], PN-91/H87026 [58]. The base alloy was made from pure alloying components in a gas furnace that was equipped with a graphite–kaolin crucible. The process of smelting carried out in this research work can be divided into 2 stages: after heating the crucible to 1100 °C, copper in the form of M1E cathode copper sheet (>99.99% purity) was loaded. In the second stage, tin (in granular form) was introduced into the molten copper. The smelting was carried out using a flux. During the smelting process, a protective coating was applied to the metal surface to limit contact between the metal and the atmosphere. The starting alloy was deoxidised with phosphorus in the form of CuP15 mordant. Once the casting melt reached a temperature of 1030 °C, it was poured into the metal dies.

The chemical composition of the starting alloy is shown in Table 1. The composition was determined using a HITACHI FOUNDRY MASTER (Hitachi, Tokyo, Japan) smart spark spectrometer during the melt process.

The alloy that was prepared for our own research was poured into metal moulds in the form of ingots; this served as a charge material for the subsequent technological tests that were conducted in a laboratory furnace.

The experimental melts were carried out in a Nabertherm furnace with a crucible capacity of 30 kg. The charge material that was used for the technological tests was the previously prepared starting alloy whose composition is presented in Table 1. In the melting process, a flux called cupuniwesal was used, and the melt was conducted under a cover of charcoal. The chemical composition of the experimental melts is shown in Table 2 and Table 3. Batches 1W–3W were alloys with different concentrations of the main alloying element (Sn). Melts 1–4 also contained alloying additives in the form of silicon, zinc, and zirconium.

From experimental Melts 1–4, castings were produced in the following technological variants:

(a) Green sand mould and core;
(b) Green sand mould, core made in CO_2_ moulding process;
(c) Mould and core made in CO_2_ moulding process;
(d) Mould and core made in CO_2_ moulding process—dried form.

Herein, the samples are marked as follows: the number (1–4) corresponds to the chemical composition, and the letter (a–d) corresponds to the type of moulding technology that was used.

### 2.2. Microstructure Analysis

For the macro- and microstructure examinations, a Nikon SMZ 745Z stereoscopic microscope and a Nikon Eclipse LV 150 metallographic microscope (Tokyo, Japan) equipped with Nis-Elements (Melville, NY, USA) image recording and analysis software ver. 3.22.15 were used.

Observations of the alloy were also conducted using a TESCAN MIRA scanning electron microscope (SEM) (Brno, Czech Republic) with an EDS Ultim Max chemical composition analysis system by Oxford Instruments (Abingdon, UK).

The metallographic samples were embedded using a Struers CitoPress-5 device (ver. 3.22.15). The prepared samples were placed on a Mecatech 234 grinding and polishing machine, where the grinding and polishing operations were performed.

### 2.3. Tightness Test

The tightness tests for the produced castings were conducted using a FORTEST T8990 device (Modena, Italy). The equipment has ambient direct pressure as the primary measure and pressure decay inside the piece under test as the second measure. We can divide the tightness test using the absolute pressure drop method into 5 stages: wait, filling, settling, test, discharge. During the “waiting” stage, the device is ready for the start signal. Stage 2 corresponds to the “filling” stage, in which the internal pneumatic circuit is operated to allow pressure to build up in the component under test. The most important stage is “settling”, which involves stabilising the pressure and temperature inside the component under test. 

During stage 4, the equipment performs a test that determines whether the test item is sufficiently airtight, based on the pressure drop inside the test item. The stage is completed by showing the pressure drop as a numerical value. The “discharge” phase is performed at the end of the test, regardless of the result.

During the test, the working pressure was set to 3 atm, and the measurement time was 60 s.

### 2.4. Mechanical Property Analysis

The mechanical properties of the tested alloy were determined through a static tensile test. The test was conducted using an INSTRON machine (Model 1115, Norwood, Mam USA). During the test, the sample was clamped between the machine’s jaws. The lower jaw of the machine was adjustable using a screw, while the upper jaw was stationary and connected to the piston. The device was connected to a computer, where a graph was generated using compatible software. Tensile tests were carried out on raw casting samples without any mechanical processing. The measurements were conducted in accordance with the applicable standards (PN-EN ISO 6892-1:2010) [59].

The hardness of the produced casting alloys was tested using an analogue hardness tester. The Brinell method that was employed involved the use of a penetrator in the form of a carbide ball with a specific diameter D (which could be 10, 2.5, 2, or 1 mm) and the application of an appropriate force F that was dependent on the chosen penetrator diameter.

## 3. Results

### 3.1. Microstructure Analysis and Phase Analysis

**Figure 1:**
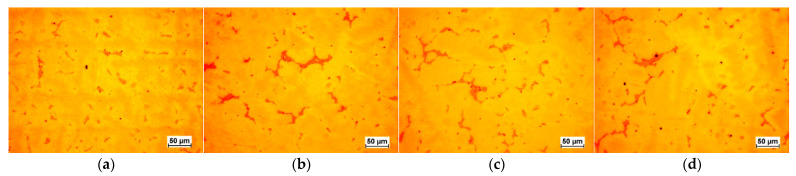
Microstructures of Sample 2 (CuSn10): (**a**) green sand mould and core; (**b**) green sand mould, core made in CO_2_ moulding process; (**c**) mould and core CO_2_ moulding process; (**d**) mould and core CO_2_ moulding process—dried mould.

**Figure 2:**
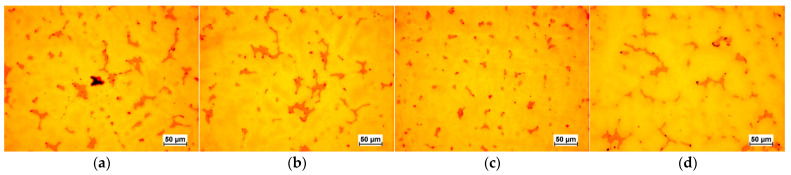
Microstructures of Sample 1 (CuSn10 + 0.04 Si): (**a**) green sand mould and core; (**b**) green sand mould, core made in CO_2_ moulding process; (**c**) mould and core CO_2_ moulding process; (**d**) mould and core CO_2_ moulding process—dried mould.

**Figure 3:**
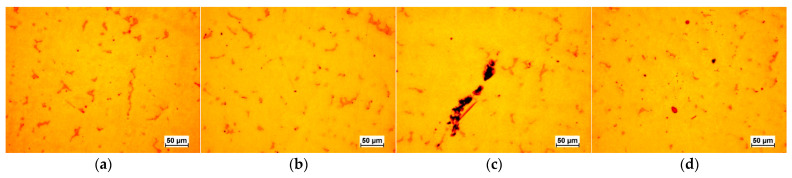
Microstructures of Sample 3 (CuSn10 + 0.23 Zn): (**a**) green sand mould and core; (**b**) green sand mould, core made in CO_2_ moulding process; (**c**) mould and core CO_2_ moulding process; (**d**) mould and core CO_2_ moulding process—dried mould.

**Figure 4:**
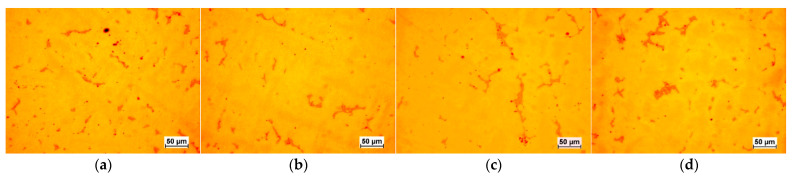
Microstructures of Sample 4 (CuSn10+ 0.02 Zr): (**a**) green sand mould and core; (**b**) green sand mould, core made in CO_2_ moulding process; (**c**) mould and core CO_2_ moulding process; (**d**) mould and core CO_2_ moulding process—dried mould.

**Figure 5:**
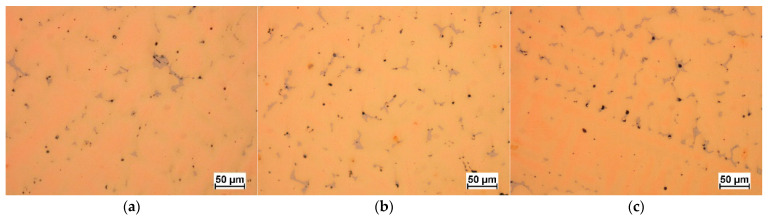
Microstructures of CuSn casting alloy green sand mould: (**a**) 1W (CuSn9); (**b**) 2W (CuSn10); (**c**) 3W (CuSn11).

The selected results of the microstructure examinations are shown in Figure 1, Figure 2, Figure 3, Figure 4 and Figure 5. The conducted research aimed to demonstrate the influence of the moulding technology: (a) sand casting mould and core (Figure 1a, Figure 2a, Figure 3a and Figure 4a); (b) sand casting mould and core CO_2_ casting process (Figure 1b, Figure 2b, Figure 3b and Figure 4b); (c) mould and core CO_2_ moulding process (Figure 1c, Figure 2c, Figure 3c and Figure 4c); and (d) mould and core CO_2_ moulding process—dried mould (Figure 1d, Figure 2d, Figure 3d and Figure 4d), as well as the chemical composition of the casting alloy on its microstructure. The introduction of tin as an alloying component (Figure 1 and Figure 5) and the introductions of silicon, zinc, and zirconium microadditives were analysed (Figure 2, Figure 3 and Figure 4, respectively). The microstructure of the cast CuSn10 bronze was two-phased and included the α (Cu) solid solution in the form of dendrites and the (α + δ) eutectoid in the interdendritic spaces (which were observed in all of the microstructures). The formation of the microstructure is the result of the influence of the chemical composition of the alloy (tin content, Figure 1 and Figure 5) and the introduced additives, silicon (Figure 2), zinc (Figure 3), and zirconium as a modifier (Figure 4). The formation of the microstructure is also influenced by the solidification rate and crystallisation. It depends, among other things, on the moulding sand used and the preparation of moulds and cores. Both the chemical composition and the conditions of heat dissipation through the mould may affect the formation of α (Cu) dendrites and eutectoid precipitates in the interdendritic spaces. Due to the compactness of the structure and thus the tightness of the castings, the amount and fineness of eutectoid precipitates (α + δ) can significantly affect the compactness of the structure and limit the formation of macroporosity. The microstructures shown in Figure 1, Figure 2, Figure 3, Figure 4 and Figure 5 do not show any significant changes. Small differences can be observed mainly in the area of fineness of eutectoid precipitates.

**Figure 6:**
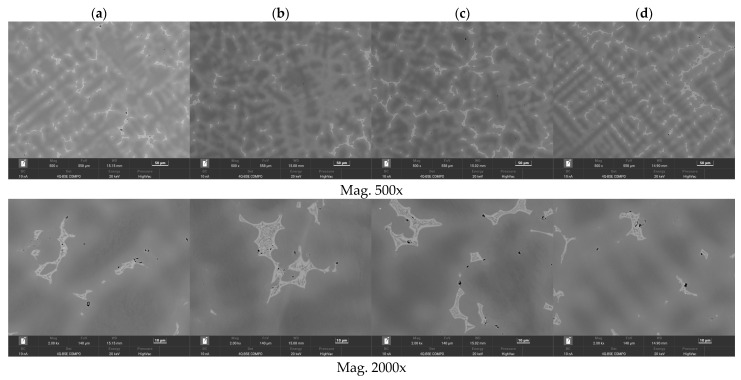
Microstructures of Sample 2 (CuSn10), (**a**) green sand mould and core; (**b**) green sand mould, core made in CO_2_ moulding process; (**c**) mould and core CO_2_ moulding process; (**d**) mould and core CO_2_ moulding process—dried mould. Measurement parameters: mag 500x and 2.00kx, FoV 140 µm, WD 14.9–15.2, BC 10 nA, Det 4Q BSE Compo, energy 20 keV, pressure HighVac.

**Figure 7:**
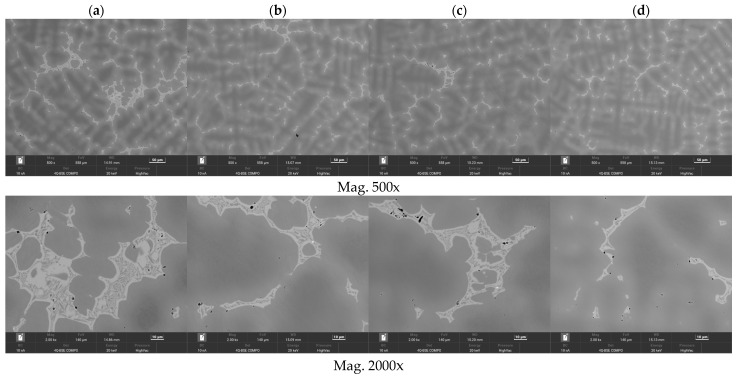
Microstructures of Sample 4 (CuSn10 + 0.02 Zr): (**a**) green sand mould and core; (**b**) green sand mould, core made in CO_2_ moulding process; (**c**) mould and core CO_2_ moulding process; (**d**) mould and core CO_2_ moulding process—dried mould. Measurement parameters: mag 500x and 2.00kx, FoV 140 µm, WD 14.9–15.2, BC 10 nA, Det 4Q BSE Compo, energy 20 keV, pressure HighVac.

**Figure 8:**
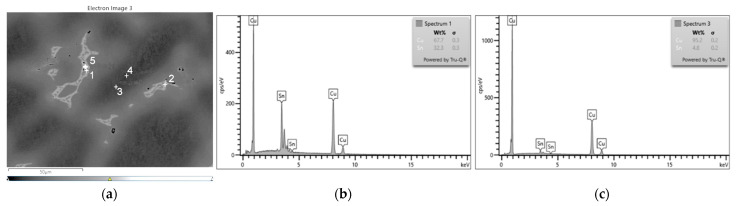
SEM-EDS analysis of microstructure and chemical composition of Sample 2 (CuSn10 alloy): (**a**) image of microstructure with points of analysis; (**b**) plot of spectra in Area 1; (**c**) plot of spectra in Area 3.

**Figure 9:**
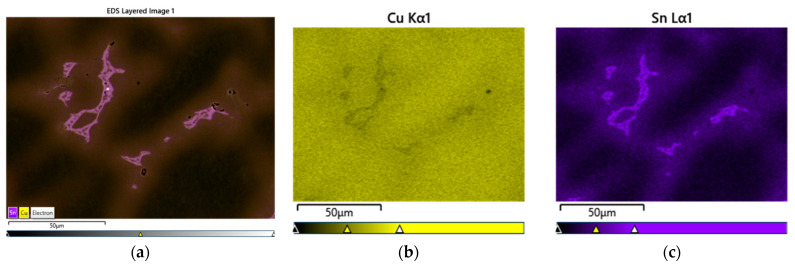
Element-distribution maps of Sample 2 (CuSn10 alloy): (**a**) submission of elements; (**b**) Cu; (**c**) Sn.

The research that was conducted using the scanning electron microscope allowed for observations of the changes in the microstructures of the CuSn10 alloy. The microstructures of the tested alloys had a dendritic characteristic. The presence of the α (Cu) solid solution and the tin-enriched eutectoid (α + δ) was observed. Casting alloys with different tin concentrations are presented in Figure 5a–c. Based on the visible microstructures (Figure 5, Figure 6, Figure 7, Figure 8 and Figure 9), it was noted that the investigated alloys contained the mentioned solid solution and eutectoid. It was noticeable that with the increased Sn concentration in the alloy, the dendrites of the α (Cu) solid solution became more distinct. It was also evident that as the tin concentration in the CuSn alloy increased, the proportion of the eutectoid (α + δ) also increased. The degree of the dendritic segregation depended on the solidification rate of the alloy in the mould. In the analysed cases, the solidification rate was the slowest for the moulding technology labelled “d” (mould and core CO_2_ moulding process—dried mould); in this technology, the dendrites were the most developed.

The research allowed for a clear observation of the dendritic microsegregation; the best shaped and most visible dendrite axes are shown in Figure 6d. Observations using a scanning electron microscope with a backscattered electron (BSE) detector enabled imaging with contrasts in the chemical composition and material density differences. In this way, the registered differences in greyscale intensity confirmed the dendritic microsegregation in the areas of the examined samples.

**Figure 10:**
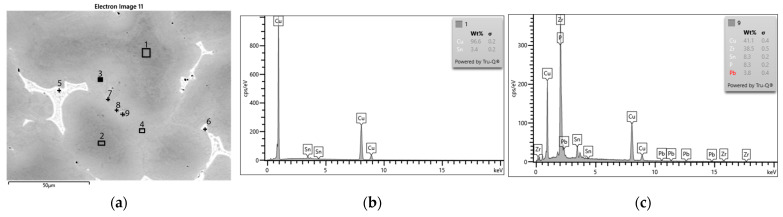
SEM-EDS analysis of microstructure and chemical composition of Sample 4 (CuSn10 + 0.02 Zr alloy): (**a**) image of microstructure with points of analysis; (**b**) plot of spectra in Area 1; (**c**) plot of spectra in Area 3.

The SEM-EDS analyses (visible in Figure 8, Figure 9 and Figure 10) and the chemical composition measurements at specific points (Table 4 and Table 5) allowed for observations of the phase structures of the alloy, regarding the assessment of the chemical composition of the individual phases and precipitations. The varied concentration of the tin and the phases that were related to the presence of zirconium and lead that were introduced into the CuSn10 alloy is noticeable.

In the microstructure of the CuSn10 alloy, the highest concentration of copper was confirmed in the dendrite axes and arms (Figure 8, Table 4, Spectra 3 and 4), while in the eutectoid (located in the interdendritic spaces), the highest concentration of tin was indicated (Figure 8, Table 4, Spectra 1 and 2). A small inclusion of lead in the eutectoid space was also observed. The distribution of the surface composition of the copper and tin is depicted in the form of a map (Figure 9). The porosities are clearly visible against the microstructure background.

**Figure 11:**
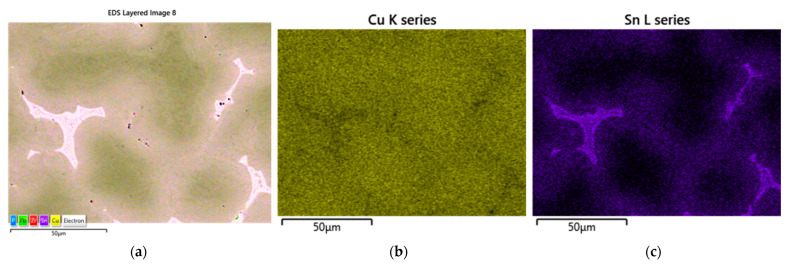

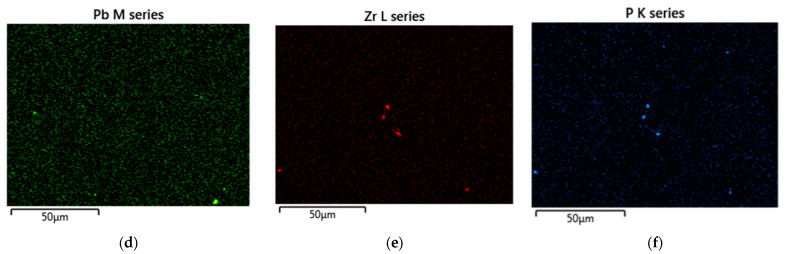
Element distribution maps of Sample 4 (CuSn10 + 0.02 Zr alloy): (**a**) submission of elements; (**b**) Cu; (**c**) Sn; (**d**) Pb; (**e**) Zr; (**f**) P.

The analyses that were conducted after the introduction of the zirconium microadditive showed a similar distribution of the solid solution and eutectoid in the sample (Figure 10, Table 5, Spectra 5–6). In the microstructure, individual zirconium precipitates were visible, occurring in the presence of phosphorus (Figure 10, Table 5, Spectra 7, 9). Zirconium and phosphorus are visible in the form of very fine precipitates visible in the interdendritic spaces. This was also confirmed by the element distribution map (Figure 11). The porosities in this sample were smaller and more widely dispersed; this could be attributed to the influence of the zirconium (even though they were not completely eliminated).

### 3.2. Tightness Test

**Figure 12:**
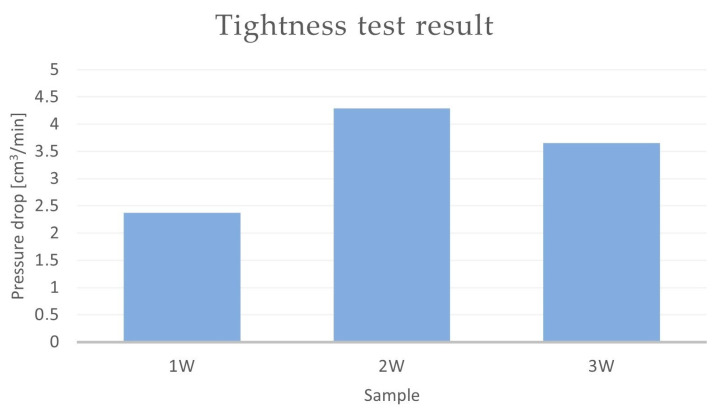
Results of tightness test—pressure drop method for Samples 1W–3W (cm^3^/min).

The castings that were made from Melts 1W–3W (Table 2) underwent mechanical processing, which was necessary in order to perform the tightness tests. The test was conducted using a FORTEST Model T8990 device that measured the pressure drop over a set time. Each test lasted for 60 s, and the working pressure was set at 3 atm. Based on the obtained data, Table 6 and Figure 12 were prepared.

According to the PN-EN 1982:2010P standard [60], the CuSn10 casting alloy can contain tin (Sn) within a range of 9 to 11%. Analysing the results of the tightness tests, Table 5, and the prepared chart that was based on them (Figure 12), it can be observed that the quantity of the main alloying element (Sn) affected the tested parameter (tightness). The conducted tests showed that the tin content, fluctuating at the lower limit of the mentioned standard (9%), had a favourable effect on the tightness of the castings (Figure 12). The concentration of tin affected the amount of eutectoid (α + δ) in the CuSn alloy, and its increased presence in the microstructure could have led to microsegregation, resulting in leakages in the casting. The pressure drop in the casting with the chemical composition labelled 1W (CuSn9) was 2.37 cm^3^/min, which was the lowest value among the three tested alloys. The highest pressure drop occurred for Sample 2W. 

**Figure 13:**
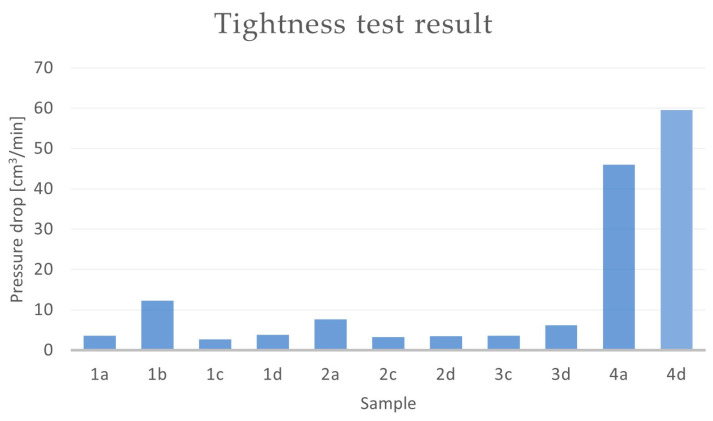
Results of tightness test—pressure drop method for Samples 1–4 (cm^3^/min).

The results of the tightness tests that were conducted on the CuSn10 bronze castings with the introduced alloying additives are presented in Table 7 and Figure 13.

Based on the obtained results, it can be concluded that the pressure drop over time depends on both the mould technology and the chemical composition of the alloy. In terms of tightness, the casting made from the alloy labelled “1” (CuSn10 + Si) and the casting made from the alloy labelled “2” (Table 3) performed the best. For the samples with chemical compositions 1 and 2, it could be observed that the choice of the moulding material also influenced the tightness of the castings. The samples with chemical composition 1 (Table 3) made in the CO_2_ moulding process (1c and 1d) exhibited smaller pressure drops over time (2.68 and 3.79 cm^3^/min, respectively) as compared to the castings that were made from the same alloy in green sand (1a and 1b) (with pressure drops of 3.65 and 12.34 cm^3^/min, respectively). A similar situation occurred for the sample labelled “2”. The castings that were made in the CO_2_ moulding process (2c and 2d) showed smaller pressure drops in the casting, with pressure drops of 3.24 cm^3^/min for Sample 2c and 3.49 cm^3^/min for Sample 2d. In the case of the same alloy in the green sand mould, the pressure drop was 7.71 cm^3^/min (Sample 2a).

The CuSn10 + Zn alloy (Sample 3) in the CO_2_ moulding process performed similarly to Sample 2. The pressure drop over time for Sample 3c was 3.65 cm^3^/min, while this value was 6.16 cm^3^/min for the casting in Sample 3d. The castings that were made in the green sand moulds were outside the scale of the measurement device.

Sample 4 (CuSn10 + Zr) exhibited the worst results in terms of the tested parameter. The pressure drop in Casting 4a (cast in green sand moulds) was 46 cm^3^/min. In the case of the casting made in the CO_2_ moulding process (dried mould), the pressure drop over time was 59.54 cm^3^/min. The CuSn10 + 0.04Zr foundry alloys in Samples 4b (green sand mould, core made in the CO_2_ moulding process) and 4c (mould and core made in the CO_2_ moulding process) were outside the established measurement scale. 

### 3.3. Mechanical Property Analysis

**Figure 14:**
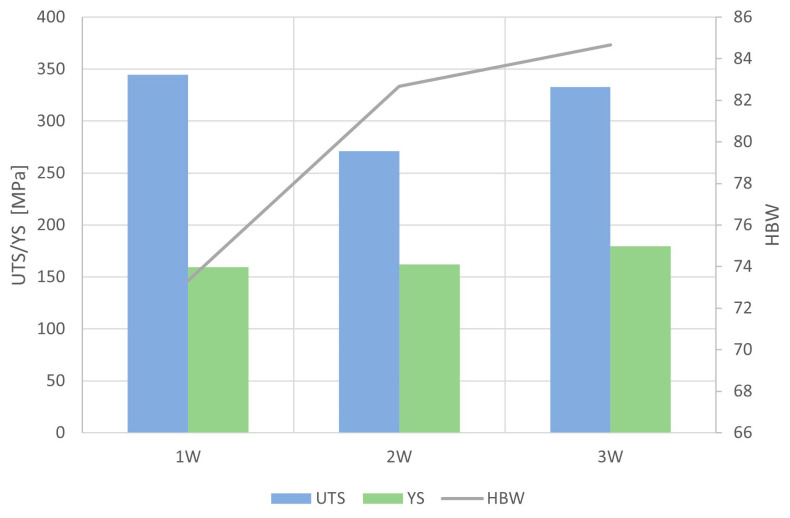
Graphical representation of mechanical properties of Samples 1W–3W.

**Figure 15:**
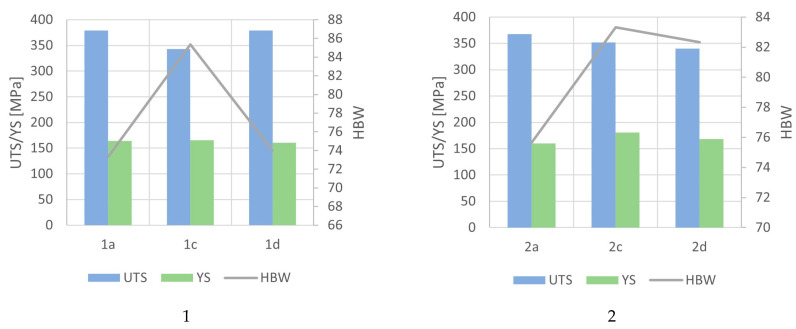

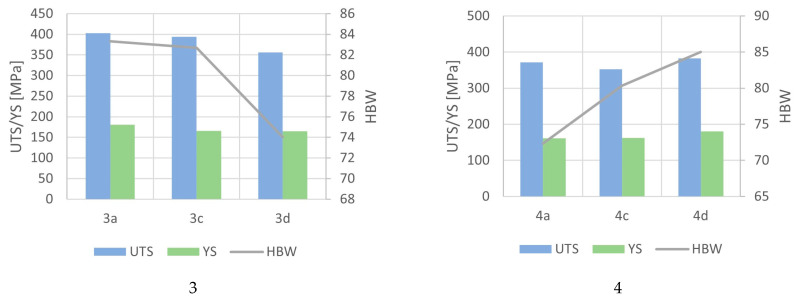
Graphical representation of mechanical properties of Samples 1–4.

Tensile strength tests were conducted using the INSTRON Model 1115 tensile testing machine. These results are presented in Table 8 and Table 9 and the charts that are shown in Figure 14 and Figure 15 were prepared based on these results.

The tin content in the tested alloy affected its mechanical properties; as the tin con-tent in the alloy increased, its hardness increased. Sample 1W (CuSn9) had the lowest hardness (73 HBW). In the case of the CuSn10 alloy (2W), the hardness of the alloy was 83 HBW. The alloy with the highest tin content (CuSn11) had a hardness of 85 HBW.

The yield strength also increased with the tin content. For Sample 1W, the yield strength was 159 Mpa. CuSn10 had a yield strength of 162 Mpa, while Sample 3W (with a tin content of 11%) had a yield strength of 180 Mpa. The ultimate tensile strength (UTS) was highest for Sample 1W (CuSn9) at 344 Mpa, while CuSn10 (2W) had the lowest UTS of 271 Mpa. Sample 3W had a tensile strength of 333 Mpa.

The tensile strength tests that were conducted on the CuSn10 alloy with added Si, Zn, and Zr are presented in Table 9 (and graphically in Figure 15).

The added alloying elements had a noticeable impact on the mechanical properties of the CuSn10 alloy. For those castings that were made in the green sand (1a–4a), the addition of zinc (Sample 3a) had the most beneficial effect on the tensile strength (with a UTS value of 402 MPa). In the case of the CuSn10, CuSn10 + Zn, and CuSn10 + Zr samples, the tensile strengths fell to within a range of 368–371 MPa.

The hardness of the tested alloys ranged from 71 to 85 HBW depending on the composition and moulding process. In the case of Sample 1 (CuSn10 + Si), the highest hardness level (83 HBW) could be observed in the casting that was made in the green sand moulding process. For this alloy (cast into the CO_2_ moulding process), the hardness was 83, and for the dried mould, this was 74 HBW. Sample 2 (CuSn10) achieved the highest hardness level when cast into the CO_2_ moulding process (83 HBW), while the dried mould yielded a hardness of 82 HBW. Those castings that were made with the green sand moulding process resulted in the lowest hardness level (measuring 76 HBW). For Sample 3 (CuSn10 + Zn), the highest hardness was achieved in the green sand moulding and CO_2_ moulding processes (both measuring 83 HBW). However, the use of a dried mould with the CO_2_ moulding process had a less favourable effect on the hardness (resulting in 74 HBW). Sample 4 (which contained zirconium as an additive) achieved the highest hardness when cast into the CO_2_ moulding process subjected to a drying process (with a hardness of 85 HBW). Those castings that were made in the green sand moulding process featured the lowest hardness level (measuring 72 HBW).

The value of the yield strength (YS) did not significantly change, neither due to the introduced alloying elements nor the moulding sand type; this remained within a range of 160 to 180 MPa, regardless of the alloy composition or moulding process. No correlation could be observed among the introduced alloying elements and the moulding sand type concerning the yield strength. 

A casting alloy from the CuSn bronze group with a similar chemical composition appearing in the standard PN-EN 1982:2017 [60] is, among others, CuSn11P-C bronze (CC481K). Apart from copper being the matrix, it contains Sn = 10.0–11.5% and P = 0.5–1.0%. In sand mould technology, the minimum values of mechanical properties are as follows: tensile strength (UTS): 250 MPa; yield strength (YS): 130; and hardness (HBW): 60. According to the international standard Unified Numbering System for Metals and Alloys (UNS), an alloy with a similar composition is C52400 (CuSn9Pb1): Sn = 9.00–11.0% and P = 0.03–035%. The mechanical properties of CuSn9Pb1 bronze reach values in the following ranges: UTS = 270–370 MPa; YS = 140–230; and HBW = 85–90.

The obtained results of strength tests of the analysed alloys exceed the minimum values of the cited alloys from the CuSn group in terms of tensile strength (UTS), yield strength (YS), and hardness (HBW).

## 4. Conclusions

The conducted research regarding the impact of the moulding sand type and the addition of silicon, zinc, and zirconium to CuSn10 bronze on its microstructure and mechanical properties allowed us to observe changes within the microstructure, the SEM-EDS results, and selected mechanical properties. The research undertaken is aimed primarily at improving the structure of shaped castings for which tightness is required. The compactness of the microstructure is the result of the solidification and crystallisation process, including the formation of shrinkage microporosities and structure discontinuities. The formation of the microstructure of the analysed bronzes results primarily from the chemical composition of the alloy. Another factor influencing the formation of the microstructure is the rate of solidification and crystallisation, which depends on the moulding sand used to prepare moulds and cores. Both the chemical composition and the conditions of heat dissipation through the mould may influence the formation of α (Cu) dendrites and eutectoid precipitates in the interdendritic spaces. Due to the compactness of the structure and therefore the tightness of the castings, the amount and fragmentation of eutectoid precipitates (α + δ) may significantly affect the compactness of the structure and limit the formation of microporosity.

Based on the research, the following conclusions can be drawn:

- The addition of zirconium to CuSn10 bronze led to the refinement of its structure.
- Silicon added to the alloy affected its fluidity and the surface quality of the castings.
- With an increase in the tin content in the alloy, the dendrites of the α(Cu) phase became more developed, and the eutectoid phase (α + δ) proportion increased in the interdendritic spaces.
- Lower tin contents in the alloy positively influenced the castings tightness due to the smaller solidification temperature range.
- The tin content in the alloy affected its hardness, and the amount of the added alloying element also affected its yield strength (YS). YS increased with higher tin concentrations in CuSn10 bronze.
- Considering the analysed technologies (1a–4d), Sample 1 (CuSn10 + Si) exhibited the best tightness parameter. The highest pressure drop in the casting could be observed for Sample 1b (green sand mould, core made in CO_2_ moulding process).
- Sample 4 (CuSn10 + Zr) showed the greatest pressure drop over time, with two measurements (4a and 4c) ending up off of the established measurement scale. The microstructures of the alloy with the zirconium microaddition displayed visible microporosity; this could have led to leakage in the tested castings.
- The moulding sand type had a minor impact on the results of the tightness tests. However, it was noticeable that most of the samples that exceeded the measurement scale were cast in the green-sand-casting process.

## Figures and Tables

**Table 1:** Base composition of CuSn10 alloy (wt%).

No.	Sn	Fe	Si	Mn	Pb	Zn	Zr	P	Cu
	(wt%)								
0	10.1	0.00	0.00	0.00	0.08	0.00	0.00	0.05	Bal.

**Table 2:** Chemical composition of experimental melts: Samples 1W–3W (wt%).

No.	Sn	Fe	Si	Mn	Pb	Zn	Zr	P	Cu
	(wt%)								
1W	9.00	0.00	0.00	0.00	0.12	0.00	0.00	0.20	Bal.
2W	10.00	0.00	0.00	0.00	0.10	0.00	0.00	0.05	Bal.
3W	11.02	0.00	0.00	0.00	0.14	0.00	0.00	0.18	Bal.

**Table 3:** Chemical composition of the experimental melts: Samples 1–4 (wt%).

No.	Sn	Fe	Si	Mn	Pb	Zn	Zr	P	Cu
	(wt%)								
1	9.30	0.00	0.04	0.00	0.08	0.00	0.00	0.21	Bal.
2	10.2	0.00	0.00	0.00	0.09	0.00	0.00	0.15	Bal.
3	10.1	0.00	0.00	0.00	0.06	0.23	0.00	0.05	Bal.
4	9.74	0.00	0.00	0.00	0.00	0.01	0.02	0.05	Bal.

**Table 4:** Chemical composition of CuSn10 alloy in micro-area from Figure 8 (wt%).

Spectrum Label	Cu	Sn	Pb	Total
Spectrum 1	67.65	32.35	-	100.00
Spectrum 2	67.36	32.64	-	100.00
Spectrum 3	95.21	4.79	-	100.00
Spectrum 4	95.69	4.31	-	100.00
Spectrum 5	37.23	13.22	49.54	100.00

**Table 5:** Chemical composition of CuSn10 + 0.02 Zr alloy in micro-area from Figure 10.

Spectrum Label	Cu	Zr	Sn	Pb	P	Total
Spectrum 1	96.58	-	3.42	-	-	100.00
Spectrum 2	96.67	-	3.33	-	-	100.00
Spectrum 3	86.93	-	13.07	-	-	100.00
Spectrum 4	87.45	-	12.55	-	-	100.00
Spectrum 5	67.17	-	32.83	-	-	100.00
Spectrum 6	67.37	-	32.63	-	-	100.00
Spectrum 7	71.88	13.68	12.32	1.66	0.46	100.00
Spectrum 8	71.53	15.97	12.50	-	-	100.00
Spectrum 9	41.11	38.54	8.31	3.76	8.28	100.00

**Table 6:** Results of tightness tests for Samples 1W–3W (cm^3^/min).

Sample	Tightness Test Result (cm^3^/min)
1W (CuSn9)	2.37
2W (CuSn10)	4.29
3W (CuSn11)	3.65

**Table 7:** Results of tightness test for Samples 1–4 (cm^3^/min).

Sample	Tightness Test Result (cm^3^/min)
1a	3.65
1b	12.34
1c	2.68
1d	3.79
2a	7.71
2c	3.24
2d	3.49
3c	3.65
3d	6.16
4a	46.0
4d	59.51

**Table 8:** Summary of strength test results of Samples 1W–3W.

Sample	UTS (MPa)	YS (MPa)	HBW
1W	344	159	73
2W	271	162	83
3W	333	180	85

**Table 9:** Summary of strength test results of Samples 1–4.

Sample	UTS (MPa)	YS (Mpa)	HBW
1a	378	164	73
1b	342	166	85
1d	379	161	74
2a	368	160	76
2b	352	180	83
2d	340	168	82
3a	402	181	83
3b	394	166	83
3d	356	165	74
4a	371	162	72
4b	352	162	80
4d	382	180	85

## Data Availability

The data that support the findings of this study are available from the corresponding authors (D.W., A.G.-K., M.P. (Marcin Papaj), P.P., M.M., M.P. (Marcin Piękoś), J.K.) upon reasonable request.

## References

[1] Engels G., Wübbenhorst H. (2007). 5000 Jahre Giessen von Metallen.

[2] Rzadkosz S. (2013). Foundry of Copper and Copper Alloys.

[3] Ottaway B.S. (2001). Innovation, production and specialization in early prehistoric copper metallurgy. Eur. J. Archaeol..

[4] Petan A., Petean I., Paltinean G.A., Filip M.R., Borodi G., Tudoran L.B. (2023). Microstructural Investigation of Some Bronze Artifacts Discovered in a Dacian Site Using Non-Destructive Methods. Metals.

[5] Chang T., Herting G., Goidanich S., Sánchez Amaya J.M., Arenas M.A., Le Bozec N., Jin Y., Leygraf C., Odnevall Wallinder I. (2019). The role of Sn on the long-term atmospheric corrosion of binary Cu-Sn bronze alloys in architecture. Corros. Sci..

[6] Zhao J., Zhang L., Du F., Yuan X., Wang P. (2022). The Microstructural Evolution of Cu-Sn-P Alloy during Hot Deformation Process. Materials.

[7] Huttunen-Saarivirta E., Kilpi L., Pasanen A.T., Salminen T., Ronkainen H. (2020). Tribocorrosion behaviour of tin bronze CuSn12 under a sliding motion in NaCl containing environment: Contact to inert vs. reactive counterbody. Tribol. Int..

[8] Calignano F., Manfredi D., Marola S., Lombardi M., Iuliano L. (2022). Production of Dense Cu-10Sn Part by Laser Powder Bed Fusion with Low Surface Roughness and High Dimensional Accuracy. Materials.

[9] Song C., Hu Z., Xiao Y., Li Y., Yang Y. (2022). Study on Interfacial Bonding Properties of NiTi/CuSn10 Dissimilar Materials by Selective Laser Melting. Micromachines.

[10] Zhang L., Li Y., Zhou R., Wang X., Wang Q., Xie L., Li Z., Xu B. (2023). First-Principles Study of the Effect of Sn Content on the Structural, Elastic, and Electronic Properties of Cu–Sn Alloys. Crystals.

[11] Rzadkosz S., Garbacz-Klempka A., Kozana J., Piękoś M., Kranc M. (2014). Structure and properties research of casts made with copper alloys matrix. Arch. Metall. Mater..

[12] Rzadkosz S., Kranc M., Garbacz-Klempka A., Kozana J., Piękoś M. (2015). Refining processes in the copper casting technology. Metalurgija.

[13] Rzadkosz S., Kozana J., Garbacz-Klempka A., Piękoś M., Cieślak W. (2014). Shaping the Microstructureand Mechanical Properties of Tin Bronzes. Arch. Foundry Eng..

[14] Kohler F., Campanella T., Nakanishi S., Rappaz M. (2008). Application of single pan thermal analysis to Cu–Sn peritectic alloys. Acta Mater..

[15] Manu K., Jezierski J., Ganesh M.R.S., Shankar K.V., Narayanan S.A. (2021). Titanium in Cast Cu-Sn Alloys—A Review. Materials.

[16] Chen C., Zhou J., Xue F., Wu Q. (2020). Elimination of liquid metal embrittlement cracks during arc cladding of tin bronze on steel sheet. Mater. Lett..

[17] Song Z., Tegus O. (2023). The Corrosion Properties of Bronze Alloys in NaCl Solutions. Materials.

[18] Wang Z., Li Y., Jiang X., Pan C. (2017). Research progress on ancient bronze corrosion in different environments and using different conservation techniques: A review. MRS Adv..

[19] Liu Y., Yu G., Cao G., Wang C., Wang Z. (2023). Characterization of corrosion products formed on tin-bronze after 29 years of exposure to Shenyang, China. J. Mater. Res. Technol..

[20] Muller J., Laïk B., Guillot I. (2013). α-CuSn bronzes in sulphate medium: Influence of the tin content on corrosion processes. Corros. Sci..

[21] (2018). Copper and Copper Alloys.

[22] Davis J.R. (2001). Copper and Copper Alloys.

[23] Zhou L., Jiang Z.H., Zhao D.G., Yu M.R., Zhao H.Y., Huang Y.X., Song X.G. (2018). Effect of Rotation Speed on the Microstructure and Mechanical Properties of Friction-Stir-Welded CuSn6 Tin Bronze. J. Mater. Eng. Perform..

[24] Sobota J., Rodak K., Nowak M. (2019). Microstructure and properties of tin bronzes produced by the SPD method. Arch. Metall. Mater..

[25] Paul C., Sellamuthu R. (2014). The Effect of Sn Content on the Properties of Surface Refined Cu-Sn Bronze Alloys. Procedia Eng..

[26] Liu Z., Zhou R., Xiong W., He Z., Liu T., Li Y. (2022). Compressive Rheological Behavior and Microstructure Evolution of a Semi-Solid CuSn10P1 Alloy at Medium Temperature and Low Strain. Metals.

[27] Slamet S., Suyitno D., Kusumaningtyas I. Effect of Composition and Pouring Temperatur of Cu-Sn on Fluidity, Density and Mechanical Properties by Investment Casting. Proceedings of the 1st International Conference on Computer Science and Engineering Technology.

[28] Audy J., Audy K. (2009). Effects of microstructure and chemical composition on strength and impact toughness of tin bronzes. MM Sci. J..

[29] Nadolski M. (2017). The evaluation of mechanical properties of high-tin bronzes. Arch. Foundry Eng..

[30] Bartocha D., Baron C., Suchoń J. (2019). The Influence of Solidification Rate on High-tin Bronze Microstructure. Arch. Foundry Eng..

[31] Paradela K.G., Baptista L.A.D.S., Sales R.C., Felipe Junior P., Ferreira A.F. (2019). Investigation of Thermal Parameters Effects on the Microstructure, Microhardness and Microsegregation of Cu-Sn alloy Directionally Solidified under Transient Heat Flow Conditions. Mater. Res..

[32] Wang X., Song J., Zhou H., Fan Z., Shi J., Chen J., Xiao K. (2023). Mechanism of dendrite segregation on corrosion behaviour of antique cast low Sn bronze. Corros. Sci..

[33] Qian Q., Baohong T., Tubing Y., Yi Z., Jingkun A., Yong L., Zhiyang Z., Jing K. (2023). Investigation of deformation comprised microstructure and precipitation of Cu–Sn–Ti alloy during hot deformation. J. Mater. Res. Technol..

[34] Li Y., He K., Liao C., Pan C. (2012). Measurements of mechanical properties of α-phase in Cu–Sn alloys by using instrumented nanoindentation. J. Mater. Res..

[35] Szajnar J., Kondracki M., Stawarz M. (2003). Modification of CuSn8 tin bronze and its influence on tin segregation. Arch. Foundry Eng..

[36] Romankiewicz F., Głazowska I. (1998). Modification of CuSn10 tin bronze. Solidif. Met. Alloy..

[37] Głazowska I., Romankiewicz F., Krasicka-Cydzik E., Michalski M. (2005). Structure of phosphor tin bronze CuSn10P modified with mixture of microadditives. Arch. Foundry.

[38] Karthik M., Abhinav J., Shankar K.V. (2021). Morphological and Mechanical Behaviour of Cu–Sn Alloys—A review. Met. Mater. Int..

[39] Popova E.N., Sudareva S.V., Romanov E.P., Dergunova E.A., Abdyukhanov I.M., Vorob’eva A.E., Elokhina L.V. (2007). Effect of alloying on the structure of bronze with enhanced tin content. Phys. Metals Metallogr..

[40] Ri K., Komkov V.G., Ri E.K. (2014). Effect of alloying elements on the physicomechanical properties of copper and tin bronze. Russ. Metall. Met..

[41] Kozana J., Garbacz-Klempka A., Piękoś M., Perek-Nowak M., Pałka P. (2021). Experimental Investigation and Thermodynamic Modeling of Influence of Nickel and Titanium Content on the Structure and Selected Properties of Tin Bronzes. Materials.

[42] Kozana J., Garbacz-Klempka A., Piękoś M., Czekaj E., Perek-Nowak M. (2018). Assessment of impact of nickel additions on tin bronzes. Arch. Foundry Eng..

[43] Taslicukur Ozturk Z., Altuğ G., Polat S., Atapek S., Türedi E. A microstructural study on CuSn10 bronze produced by sand and investment casting techniques, XXI. Proceedings of the International Conference on Metallurgy and Materials.

[44] Kadhim H.A., Abed I.J. (2021). Investigation Wear Behaviour of Tin Bronze Alloy Prepared by Different Casting Techniques. IOP Conf. Ser. Mater. Sci. Eng..

[45] Li Z., Zhao G., Wang H., Gao G., Chen S., Yang D., Fan Y., Zhang G., Xu H. (2021). Microstructure and tribological behaviors of diffusion bonded powder sintered Cu–Sn based alloys. Mater. Res. Express.

[46] Shang Q., Yu A., Wu J., Shi C., Niu W. (2017). Influence of heat affected zone on tribological properties of CuSn6 bronze laser dimple textured surface. Tribol. Int..

[47] Shi Z., Xu H., Zhang G., Liu Y., Ren X. (2023). Effect of Bi Content on the Microstructure, Mechanical and Tribological Properties of Cu-Sn Alloy. Materials.

[48] So S.-M., Kim K.-Y., Lee S.-J., Yu J., Lim H.-A., Oh M.-S. (2020). Effects of Sn content and hot deformation on microstructure and mechanical properties of binary high Sn content Cu–Sn alloys. Mater. Sci. Eng. A.

[49] Li Y.S., Wang D.W. (2014). Standard Simple of Tin Bronze for Casting Develepment. Adv. Mater. Res..

[50] Baumeister G., Buqezi-Ahmeti D., Glaser J., Ritzhaupt-Kleissl H.-J. (2011). New approaches in microcasting: Permanent mold casting and composite casting. Microsyst Technol.

[51] Sergejevs A., Kromanis A., Ozolins J., Gerins E. (2016). Influence of Casting Velocity on Mechanical Properties and Macro-Structure of Tin Bronzes. Key Eng. Mater..

[52] Jones T., Strachan R., Mackie D., Cooper M., Frame B., Vorstius J. (2021). Computational Fluid Dynamic Simulations of solidification for Improve speed of Continuous Cast copper. Eng. Sci. Technol..

[53] Sato T., Hirai Y., Kobayashi T. (2017). Development of Lead-Free Bronze with Sulfide Dispersion for Sliding Applications. Inter. Met..

[54] Bazhenov V.E., Titov A.Y., Shkalei I.V., Sannikov A.V., Nikitina A.A., Plisetskaya I.V., Bazlov A.I., Mezrin A.M. (2021). Effect of the Cooling Rate on the Microstructure and Properties of C92900 Bronze. Russ. J. Non-Ferr. Met..

[55] Schmidt R.F., Schmidt D.G. (1997). Selection and Application of Copper Alloy Casting. ASM Handbook Volume 2: Properties and Selection: Nonferrous Alloys and Special-Purpose Materials.

[56] Schmidt R.F., Schmidt D.G., Sahoo M. (1998). Copper and Copper Alloys. ASM Handbook Volume 15: Casting.

[57] (2017). Copper and Copper Alloys—Ingots and Castings.

[58] PN-91/H87026. https://kigema.com/bronze-brass-standards/ba93-pn-91-h-87026/.

[59] (2010). Metals—Tensile Test—Part 1: Room Temperature Test Method.

[60] (2017). Copper and Copper Alloys—Pigs and Castings.

